# Duopoly Price Competition in Wireless Sensor Network-Based Service Provision

**DOI:** 10.3390/s18124422

**Published:** 2018-12-14

**Authors:** Xianwei Li, Liang Zhao, Zhenyu Zhou, Bo Gu, Guolong Chen, Fanyong Cheng, Haiyang Zhang

**Affiliations:** 1 School of Information Engineering, Suzhou University, Suzhou 234000, China; xianweili@fuji.waseda.jp (X.L.); cglbox@sina.com (G.C.); 2 Global Information and Telecommunication Institute, Waseda University, Tokyo 169-0051, Japan; 3 School of Computer Science, Shenyang Aerospace University, Shenyang 110136, China; lzhao@sau.edu.cn; 4 School of Electrical and Electronic Engineering, North China Electric Power University, Beijing 102206, China; 5 School of Intelligent Systems Engineering, Sun Yat-sen University, Guangzhou 510275, China; 6 Fujian Provincial Key Laboratory of Information Processing and Intelligent Control, Minjiang University, Fuzhou 350108, China; b12090031@hnu.edu.cn; 7 School of Environment and Geomatics Engineering, Suzhou University, Suzhou 234000, China; seazhang188@126.com

**Keywords:** WSNs, service provision, noncooperative strategic game, Stackelberg game

## Abstract

The Internet of Things (IoT) is emerging as a new communication paradigm and has attracted a significant amount of attention from both academic and engineering communities. In this paper, we consider an IoT market where three roles exist: Wireless Sensor Networks (WSNs), two service providers (SPs) and end users. The WSNs are responsible for sensing and providing data to the two SPs. Based on the sensed data from WSNs, the two SPs compete to provide services to the end users. We model the relationship between the two SPs and end users as a two-stage Stackelberg game, where the two SPs set the prices for their services firstly, and then the end users decide which SP to choose. Specifically, we consider two price-competition scenarios of the two SPs, which are engaged in two games, one is a noncooperative strategic game (NSG) where the two SPs set the prices for services simultaneously, the other is a Stackelberg game (SG) where SP1 who sets the price first is the leader and SP2 who sets the price after is the follower. Each user decides whether and which SP to purchase services from based on prices and service rates. An equilibrium is achieved in each of the two scenarios. Numerical results are conducted to verify our theoretical analysis.

## 1. Introduction

**Figure 1:**
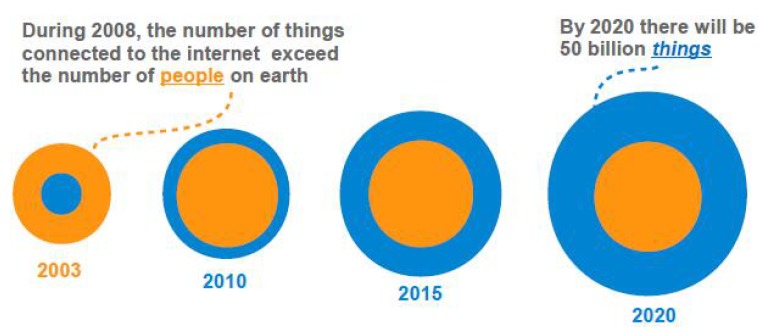
The growing number of “things” connected to the Internet [1,10].

The Internet of Things (IoT) is emerging as a new communication paradigm and has attracted a significant amount of attention from both academic and engineering communities. IoT has been widely applied in a large number of areas, such as health care, transportation, environmental monitoring, and smart buildings [1,2,3]. It is estimated that the number of smart objects in homes, offices, factories, and vehicles will reach 50 billion by 2020 compared with 12.5 billion in 2010 [4]. As shown in Figure 1, the things that are connected through Internet had passed the population of people on earth by 2008 [1]. According to a report from Cisco, the amount of data generated by IoT devices will reach 600 ZB data per year by 2020 [5]. IoT devices, such as Apple Watch and Google Glass, need to transmit their sensed data to the network service provider or the cloud service provider for data processing and analysis [6]. Although smart objects are becoming increasingly pervasive and ubiquitous in our daily life, the market models for the services related to these objects is still in its infancy [4,7]. The emergence of IoT has brought about new business models and markets [8]. From the economic perspective, the core benefit of the IoT is to create more revenues for the business [9]. Thus, understanding the business is of paramount importance. In addition, there may exist many service providers (SPs) who compete to provision IoT services to users leading to a competitive IoT market. Therefore, the successful deployment of sensor-based services needs a well understanding of both the market structure and service pricing schemes.

Software-defined networks (SDN) is an active research area in next generation networks, and it is recognized as a foreseeable application in IoT [11,12]. In SDN, network intelligence, who is usually logically centralized in SDN controllers, can monitor and control network states flexibly by OpenFlow protocols [12]. By using OpenFlow protocol and SDN controller, the owners of network infrastructures, such as the network service providers owning WSNs, could programmatically assign the virtual network services to virtual service providers in a fine-grained way [12].

IoT has received a significant amount of attention in recent years and an enormous number of efforts have been devoted to it. In [1], Miorandi et al. presented a survey of concept, development, research context and challenges, and applications for IoT. In [9], Niyato et al. proposed a new pricing scheme for services of SPs offered to end users in the IoT context. Their bundling strategy model allows multiple SPs to cooperate with each other to form a coalition and deliver their IoT services as a bundle to the end users, which can attract more end users and obtain more revenue. In [13], Niyato et al. studied IoT services from the economic aspects, which have a great impact on the successful applications of IoT. They proposed a game theoretic model, which considers both the substitute and complementary services, for price competition of IoT services provision.

In [4], Guijarro et al. proposed and analyzed a business model consisting of WSNs, multiple SPs and end users. These SPs lease sensed data as services from WSNs and compete to provide services to the end users in an oligopoly IoT market. In [14], Guijarro et al. analyzed a business model for a service platform which acts as mediator between WSNs and end users. They proposed two payment methods to solve the profit maximization problem of the service platform. Similar to [14], in [7], Guijarro et al. proposed a business model for the provision of IoTs services through a Brokering platform that intermediates between WSNs and the end users . They proposed a payment method to solve the profit maximization problem of the Brokering platform. In [8], the authors proposed a business model, which is composed by WSNs, multiple SPs and end users, for the provision of WSNs-based services. They studied the price competition between two SPs providing services to a common pool of end users. In [15], the author studied two SPs with their own private sensor networks competing to provision WSN-based services. A game-based services price decision (GSPD) model is proposed in cyber-physical systems, where service organizers collect service from service entities and provide better combined services to users [16]. Although the system model is similar to our work, we analyze the competition between SPs, while the authors in [16] mainly focus on the price competitive relationship among service owners.

Price competition is widely studied in the literature. In [17], Ren et al. studied price competition in a femtocell communications market between two network service providers, and they analyzed whether the entrant network service provider to enter the network market or not and which spectrum sharing technology to adopt to maximize its revenue. However, this work only considered the SG case. In [18], Zhang et al. studied time-dependent pricing in a duopoly network market, where two network service providers compete to attract a common pool of users, but they only considered simultaneous competition case. Although the authors in [19] studied two competition scenarios, cost factor and users’ heterogeneous preferences for network services were not taken into account. In [20], the authors proposed a QoE-ensured price-competition model for emerging mobile networks. However, they only considered one competition scenario, and they did not analyze the effects of different competition scenarios.

Our system model is mainly inspired by [8,15] as well as [4,7]. We differ from them in the following aspects. First, Sun et al. [8,15] only considered simultaneous-play competition between two sensor SPs, that is, the two SPs set the prices for their services simultaneously. However, our work not only considered simultaneous-play competition but also analyzed the SG scenario where the two SP set the prices for their services sequentially. In particular, in [8], the authors studied price competition in a duopoly scenario, where the two SPs buy resources from WSNs, offer the composed useful services to users, and set the prices for their WSN-based services simultaneously. In [8], the authors incorporated the reservation prices in the user utility functions, which are not considered in our work. In [15], the authors analyzed the relationship among two sensor SPs, the network operator, and the end users, while we mainly considered the relationship between the SPs and users. Based on the Logit discrete choice model related to the quality of the collected data and the subscription price, the two SPs decide to subscribe or not to the network operator to upload the collected sensing data that are to be processed by the network operator, and then provide sensor-data-based services to users. The utility functions of the SPs and users in [15] are different from us. Second, Guijarro et al. [4] studied price competition in the oligopoly IoT market where there are more than two SPs, and they only analyzed the simultaneous-play competition. Third, Guijarro et al. [7] only analyzed the monopoly IoT market without considering the competition between SPs.

There are also some works proposing business models in cloud service provision, where SPs compete to provide services for users by leasing resources from infrastructure providers, such as [21,22,23]. However, the cloud service provision models are different from us and they only consider one competition case.

This paper tries to understand the business model of Wireless sensor networks (WSNs)-based service provision, which is recognized as a likely scenario for the realization of IoT [4,24]. In particular, we propose a business model and analyze the duopoly price competition between two SPs. The business model consists of WSNs, who are responsible for gathering sensing data; the two SPs, who pay to buy the sensing data from WSNs and provide services to end users; and the end users, who subscribe to services from one of the two SPs.

Our main contributions are summarized as follows:

- We study price competition in an IoT market, where two SPs compete to provide WSNs-based services to a common of end users. As different types of end users generally have different requirements for the quality of services [25], we take end users’ different willingness-to-pay (WTP) for service quality into consideration.
- We model the relationship between the two SPs and end users in the IoT market as a two-stage Stackelberg game (SG), where the two SPs set the prices for their services in the first stage. Then, based on the qualities and prices of the offered services of the two SPs, the end users make decisions to subscribe or not to services from one of the two SPs in the second stage. We note that although in [15] the relationship between the network operator and the SPs, and the relationship between SPs and users are both modelled as a two-stage SG, the solution methods in each stage are different from our work.
- In SG, the two SPs set the prices for their services sequentially, while in noncooperative strategic game (NSG), the two SPs set the prices for their services simultaneously. Different from many of the existing works that only consider SG, in this paper, we consider two competition scenarios between the two SPs, i.e., a NSG and SG, respectively. A unique equilibrium is achieved in each of the two scenarios.
- Numerical results are performed to verify the theoretical analysis. Our numerical analysis show that both SPs can obtain more profits if they offer services with better qualities. SP1 can attract more users in the SG scenario while SP2 can attract more user in the NSG scenario, and both SPs get more profits in the SG scenario. We also present the analysis on cost factors to show how they impact the profits of the two SPs.

The rest of the paper is organized as follows. The system model is introduced in Section 2. We analyze the duopoly IoT market in Section 3. Numerical results are conducted to verify our analysis and the results are shown in Section 4. Finally, we conclude this paper and show future works in Section 5.

## 2. System Model

**Figure 2:**
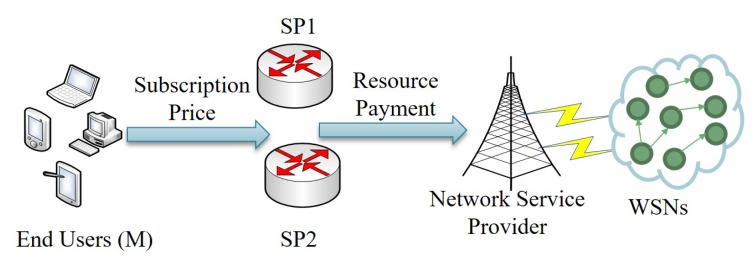
The system model.

The system model used in this paper is shown in Figure 2, which is motivated by [8,15]. The business model is composed by WSNs, two SPs and *N* end users. The WSNs owned by a network service provider are responsible for processing and providing data to the two SPs [10,26]. The two SPs, such as Apple and Google, transmit the sensed data to the network work service provider for further processing and analysis, and pay to the NSP to buy the processed and analyzed data. Then, the two SPs compete to provide data as services to the end users. The users choose to subscribe to the services according to the prices and qualities of these services offered by SPs.

We assume the data rate of the services provided by SP1 and SP2 are $R_{1}$ and $R_{2}$ (measured by the number of bits per second), respectively, and the price per data rate paid by the two SPs to the network service provider is μ. The network service provider can provide the sensed data with different QoS to the two SPs by adopting the paradigm of SDN [11,12,27] . The data rate reflects the quality of the sensed data services that SPs provide [9,28]. We use the following affine function to denote μ [8]:

(1) $$\mu =\alpha +\beta (R_{1}+R_{2}),$$

where α and β are non-negative constant values. This function implies the fact that the price of per unit resource will become higher as the aggregate data rate increases.

For SP*i*, i=1,2, its profit can be expressed as

(2) $$\pi_{i}=N_{i}p_{i}-\mu R_{i}=N_{i}p_{i}-R_{i}\left[\alpha +\beta \left(R_{1}+R_{2}\right)\right],i=1,2.$$

where $N_{i}$ is the number of end users that subscribes to the services of SP*i*.

The end users are interested in paying to use a range of services provided by the two SPs. We assume that the end users have heterogeneous preferences for the quality of the WSNs-based services. The end users’ heterogeneous preferences are characterized by their WTP, denoted by θ, which is assumed to be uniformly distributed in [0, 1] with probability distribution function (PDF) f(·) and cumulative distribution function (CDF) F(·). The uniform distribution is widely used in the literature [8,21] and one of the main reasons for the assumption of uniform distribution is for convenience of analysis. A higher value of θ means this user has higher requirement for the quality of the service.

Following [8,9], the utility that the end user k,k∈{1,2,…,N} gets from the service of SP*i*, i=1,2, is assumed to depend on the data rate $R_{i}$, which is denoted as

(3) $$U_{k,i}=\theta_{k}R_{i}-p_{i},\;i=1,2.$$

where $\theta_{k}$ is user *k*’s WTP and $p_{i}$ is the service subscription price of SP*i*.

**Remark** **1.** *It is important to note that the two SPs the flat-fee pricing schemes, which allow users to freely use services during a period. Therefore, the unit of the prices of the two SPs can be $. This kind of pricing scheme is widely in the wireless networks and cloud computing context.*

## 3. Duopoly Competitive IoT Market

In this section, we analyze a duopoly IoT market where two SPs compete by setting optimal prices for their services to maximize their profits. We consider two competition scenarios: NSG and SG. The NSG scenario corresponds to the practical IoT market where two SPs set the prices for their provided services simultaneously, while the SG scenario is the case that an entrant SP2 plans to set the prices for its services in an IoT market whose incumbent SP1 has set the prices for its services with better quality.

**Figure 3:**
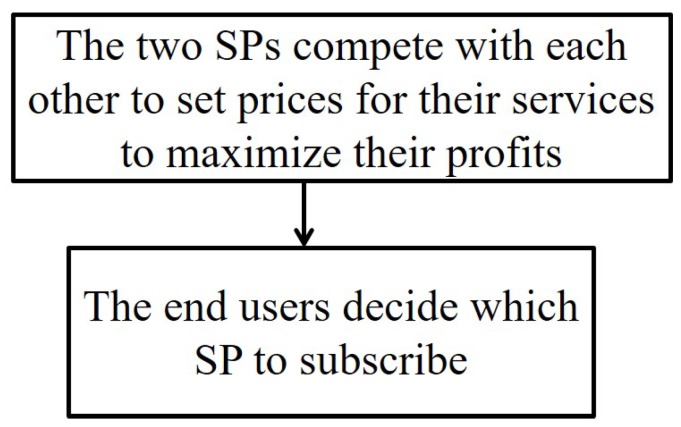
The two-stage Stackelberg game.

Based on quality of services and the subscription prices of the two SPs, the end users will make decisions as to which SP to subscribe to maximize their utilities. The relationship between SPs and users is modelled as a two-stage Stakelberg game [22], as shown in Figure 3, where the two SPs set the prices of their services in Stage I, and end users will make their joining decisions in Stage II. We solve the Stakelberg game by employing the backward induction method [29].

We note that there are two types of competition scenarios: static scenario and dynamic scenario. For ease of analysis, we only consider the static scenario in this paper, and the dynamic scenario is left for the future work.

Given the data rates $R_{1}$ and $R_{2}$, and subscription prices $p_{1}$ and $p_{2}$ of the two SPs, the end users will decide which SP to choose to maximize their utilities. We first consider three types of end users, $\theta_{1}$, $\theta_{2}$ and $\theta^{*}$, such that $U_{1,1}\left(\theta_{1},p_{1}\right)$ = 0, $U_{2,2}\left(\theta_{2},p_{2}\right)$ = 0, and $U_{k,1}\left(\theta^{*},p_{1}\right)$ = $U_{k,2}\left(\theta^{*},p_{2}\right)$, from which we have

(4) $$\theta_{1}=\frac{p_{1}}{R_{1}},$$

(5) $$\theta_{2}=\frac{p_{2}}{R_{2}},$$

(6) $$\theta^{*}=\frac{p_{1}-p_{2}}{R_{1}-R_{2}}.$$

For $R_{1}=R_{2}$, if $p_{1}\geq p_{2}$, then $U_{1}\leq U_{2}$, all users will choose to subscribe to SP1, and if $p_{1}<p_{2}$, then $U_{1}>U_{2}$, all users will choose to subscribe to SP2. For $R_{1}>R_{2}$, if $p_{1}\leq p_{2}$, then $\theta^{*}\leq 0$. In this case, $U_{1}>U_{2}$ and all users will choose to subscribe to SP1. For $R_{1}>R_{2}$, if $p_{1}>p_{2}$, then $\theta^{*}>0$. We discuss the joining decision policy of users of this case.

The type *k* user will make the following joining decision policy:

- It will join SP1 if $U_{k,1}\left(\theta_{k},p_{1}\right)>U_{k,2}\left(\theta_{k},p_{2}\right)$, and $U_{k,1}\left(\theta_{k},p_{1}\right)>0$, which requires $\theta_{k}>\theta^{*}$ and $\theta_{k}>\theta_{1}$;
- It will join SP2 if $U_{k,2}\left(\theta_{k},p_{2}\right)>U_{k,1}\left(\theta_{k},p_{1}\right)$, and $U_{k,2}\left(\theta_{k},p_{2}\right)>0$, which requires $\theta_{2}<\theta_{k}<\theta^{*}$;
- It will join neither of the two SPs if $U_{k,1}\left(\theta_{k},p_{1}\right)<0$, and $U_{k,2}\left(\theta_{k},p_{2}\right)<0$, which requires $\theta_{k}<\theta_{1}$ and $\theta_{k}<\theta_{2}$.

Based on the above joining decision policy, the fraction of end users that choose SP1 and SP2 are respectively denoted as

(7) $$F_{1}=\int_{max\{\theta_{1},\theta^{*}\}}^{1}f(\theta )d\theta ,$$

(8) $$F_{2}=\int_{\theta_{2}}^{\theta^{*}}f(\theta )d\theta ,$$

Based on Equations (7) and (8), we get the following results:

**Proposition** **1.** *For a given pair of prices ($p_{1}$, $p_{2}$), there exists a unique pair fraction of end users $F_{1}$ and $F_{2}$ that choose SP1 and SP2 respectively, such that*

*(1)*  If $\theta_{1}>\theta^{*}$, which leads to $\frac{R_{1}}{p_{1}}>\frac{R_{2}}{p_{2}}$, from which we get $\theta^{*}<\theta_{1}<\theta_{2}$. According to Equations (7) and (8), we have $F_{1}=1-F\left(\theta_{1}\right)$ and $F_{2}=0$;
*(2)*  If $\theta_{1}<\theta^{*}$, which leads to $\frac{R_{1}}{p_{2}}<\frac{R_{2}}{p_{1}}$, from which we get $\theta_{2}<\theta_{1}<\theta^{*}$. According to Equations (7) and (8), we have $F_{1}=F\left(\theta^{*}\right)$ and $F_{2}=F\left(\theta^{*}\right)-F\left(\theta_{2}\right)$;

Case 1 is the monopoly IoT market of the SP1 and case (2) is the duopoly IoT market where the two SPs coexist. As we mainly focus on the analysis of the duopoly IoT market, therefore, we only consider the case 2. For the $R_{1}>R_{2}$ case, from the above discussions, the number of end users with SP1 and SP2 in equilibrium can be denoted as follows,

(9) $$N_{1}=NF_{1}=N\left(1-\frac{p_{1}-p_{2}}{R_{1}-R_{2}}\right),$$

(10) $$N_{2}=NF_{2}=N\left(\frac{p_{1}-p_{2}}{R_{1}-R_{2}}-\frac{p_{2}}{R_{2}}\right).$$

We discuss and describe the joining decision policy of end users in Appendix A.

For $R_{1}<R_{2}$, if $p_{1}\geq p_{2}$, then $\theta^{*}\leq 0$. In this case, $U_{2}>U_{1}$ and all users will choose to subscribe to SP2. For $R_{1}<R_{2}$, if $p_{1}<p_{2}$, we can follow the similar analysis procedure to the $R_{1}>R_{2}$ case to get the results. When $R_{1}<R_{2}$, the number of end users with SP1 and SP2 in equilibrium can be denoted as follows,

(11) $$N_{1}=NF_{1}=N\left(\frac{p_{1}-p_{2}}{R_{1}-R_{2}}-\frac{p_{1}}{R_{1}}\right),$$

(12) $$N_{2}=NF_{2}=N\left(1-\frac{p_{1}-p_{2}}{R_{1}-R_{2}}\right).$$

Based on the equilibrium number of the end users in Equations (9)–(12), the two SPs will compete to maximize their profits, which can be formulated as the following one-shot game:

- **Players**: SP1 and SP2 are the two players in the game;
- **Strategies**: SP1 and SP2 determine subscription prices $p_{1}$ and $p_{2}$, respectively;
- **Payoff**: The profits of SPs, which will be defined later by $\pi_{1}=p_{1}N_{1}$ and $\pi_{2}=p_{2}N_{2}$.

### 3.1. Nash Equilibrium in the Duopoly IoT Market

A pair of prices ($p_{1}^{*}$, $p_{1}^{*}$) is said to be a Nash Equilibrium if they satisfy [30]:

(13) $$\pi_{1}\left(p_{1}^{*},\;p_{2}^{*}\right)\geq \pi_{1}\left(p_{1},\;p_{2}^{*}\right),\;\forall p_{1}\geq 0,$$

(14) $$\pi_{2}\left(p_{1}^{*},\;p_{2}^{*}\right)\geq \pi_{1}\left(p_{1}^{*},\;p_{2}\right),\;\forall p_{2}\geq 0.$$

In the Nash Equilibrium, any SP cannot change its price unilaterally to increase its profit. That is equivalent to saying the Nash Equilibrium price is the optimal price that a SP can achieve in an IoT market when SPs compete with each other. In the Nash Equilibrium, both SPs get the optimal profits.

### 3.2. Noncooperative Strategic Game (NSG)

We analyze the NSG scenario [19] where the two SPs compete by setting the prices of their services simultaneously to maximize their profits. It is important to note that we only consider $R_{1}>R_{2}$ in NSG scenario, as the two SPs set the prices for their services simultaneously in this scenario. For the $R_{1}<R_{2}$ case, we can get the similar results. The NSG scenario corresponds to the practical IoT market where two SPs with different quality of services begin to set the prices for their offered services simultaneously. Based on the number of end users in equilibrium $N_{1}$ and the given subscription price $p_{1}$, the profit optimization problem of SP1 is formulated as **Problem1:**

(15) $$\begin{array}{c} \max_{p_{1}}\pi_{1}\\ s.t.\;p_{1}\geq 0 \end{array}$$

where $N_{1}$ is given in Equation (9) and $\pi_{1}$ is denoted as

(16) $$\begin{array}{cc} \pi_{1} & =N_{1}p_{1}-R_{1}\left[\alpha +\beta \left(R_{1}+R_{2}\right)\right]\\ & =N\left(1-\frac{p_{1}-p_{2}}{R_{1}-R_{2}}\right)p_{1}-R_{1}\left[\alpha +\beta \left(R_{1}+R_{2}\right)\right]. \end{array}$$

Similarly, given the number of end users in equilibrium $N_{2}$ and the given subscription price $p_{2}$, the profit optimization problem of SP2 in NSG scenario is formulated as **Problem2:**

(17) $$\begin{array}{c} \max_{p_{2}}\pi_{2}\\ s.t.\;p_{2}\geq 0 \end{array}$$

where $N_{2}$ is given in Equation (10) and $\pi_{2}$ is denoted as

(18) $$\begin{array}{cc} \pi_{2} & =N_{2}p_{2}-R_{2}\left[\alpha +\beta \left(R_{1}+R_{2}\right)\right]\\ & =N\left(\frac{p_{1}-p_{2}}{R_{1}-R_{2}}-\frac{p_{2}}{R_{2}}\right)p_{2}-R_{2}\left[\alpha +\beta \left(R_{1}+R_{2}\right)\right]. \end{array}$$

By solving the above two problems respectively, we have the following results, which are proved in Appendix B.

**Proposition** **2.** *There exists a unique Nash Equilibrium price pair ($p_{1}^{n}$, $p_{2}^{n}$) in the NSG scenario.*

Based on Proposition 2, we have the following corollary.

**Corollary** **1.** *The profits of the two SPs in the NSG scenario are denoted as:*

(19) $$\pi_{1}^{n}=p_{1}^{n}N_{1}^{n}-R_{1}\left[\alpha +\beta \left(R_{1}+R_{2}\right)\right],$$

(20) $$\pi_{2}^{n}=p_{2}^{n}N_{2}^{n}-R_{2}\left[\alpha +\beta \left(R_{1}+R_{2}\right)\right].$$

*where $N_{1}^{n}$ and $N_{2}^{n}$ the number of users that choose of SP1 and SP2 in NSG scenario for the $R_{1}>R_{2}$ case.*

### 3.3. Stackelberg Game (SG)

We next analyze the strategic interaction between two SP1 and SP2 which is modelled as a SG [29,31]. We first consider the $R_{1}>R_{2}$ case, and then analyze the $R_{1}<R_{2}$ case. Under the condition that $R_{1}>R_{2}$, the SG scenario corresponds to the practical case that the entrant SP2 plans to set the prices for its offered services in an IoT market whose incumbent SP1 has set the prices for its services with better quality of service. We assume that SP1 is the game leader and SP2 is the game follower. SP1 first sets subscription price to maximize its profits and SP2 sets subscription price by anticipating SP1’s response. Then, the equilibrium prices of the two SPs in the SG scenario are obtained by using the backward induction method.

Based on the subscription price of SP1, SP2 sets subscription price to maximize its profits, which is formulated as **Problem3:**

(21) $$\begin{array}{c} \max_{p_{2}}\pi_{2}\\ s.t.\;p_{2}\geq 0 \end{array}$$

where $N_{2}$ is given in Equation (10) and $\pi_{2}$ is denoted in Equation (18).

Given $N_{1}$ and the subscription price $p_{1}$, the profit optimization problem of SP1 is formulated as **Problem4:**

(22) $$\begin{array}{c} \max_{p_{1}}\pi_{1}\\ s.t.\;p_{1}\geq 0 \end{array}$$

where $N_{1}$ is given in Equation (9) and $\pi_{1}$ is denoted in Equation (17).

By solving Equations (21) and (22), we get the following results, which are proved in Appendix C.

**Proposition** **3.** *Under the condition that $R_{1}>R_{2}$, there exists a unique Nash Equilibrium price pair ($p_{1}^{s}$, $p_{2}^{s}$) in the SG scenario in the IoT market.*

Accordingly, we get the following corollary:

**Corollary** **2.** *Under the condition that $R_{1}>R_{2}$, the profits of SP1 and SP2 in the SG scenario are denoted as:*

(23) $$\pi_{1}^{s}=p_{1}^{s}N_{1}^{s}-R_{1}\left[\alpha +\beta \left(R_{1}+R_{2}\right)\right],$$

(24) $$\pi_{2}^{s}=p_{2}^{s}N_{2}^{s}-R_{2}\left[\alpha +\beta \left(R_{1}+R_{2}\right)\right].$$

*where $N_{1}^{s}$ and $N_{2}^{s}$ are the number of users that respectively choose SP1 and SP2 in SG scenario for the $R_{1}>R_{2}$ case.*

For the case $R_{1}<R_{2}$, we can get the following results by following the similar analysis procedure to the $R_{1}>R_{2}$ case, which are proved in Appendix D.

**Proposition** **4.** *Under the condition that $R_{1}<R_{2}$, there exists a unique Nash Equilibrium price pair ($p_{1}^{s}$, $p_{2}^{s}$) in the SG scenario in the IoT market.*

**Corollary** **3.** *Under the condition that $R_{1}<R_{2}$, the profits of SP1 and SP2 in the Stackelberg game scenario are denoted as:*

(25) $$\pi_{1}^{s}=p_{1}^{s2}N_{1}^{s2}-R_{1}\left[\alpha +\beta \left(R_{1}+R_{2}\right)\right],$$

(26) $$\pi_{2}^{s}=p_{2}^{s2}N_{2}^{s2}-R_{2}\left[\alpha +\beta \left(R_{1}+R_{2}\right)\right].$$

*where $N_{1}^{s2}$ and $N_{2}^{s2}$ are the number of users that respectively choose SP1 and SP2 in SG scenario for the $R_{1}<R_{2}$ case.*

## 4. Simulation Results

In this section, we present simulations results to analyze and discuss our analysis in the previous sections. More in detail, we measure how users’ joining decision policy, and SPs’ equilibrium prices and profits vary with different parameters in the considered two competition scenarios.

### 4.1. Parameter Setting

We apply the default parameters of duopoly IoT market as follows: for the $R_{1}>R_{2}$ case, $R_{1}=50$, $R_{2}=20$, for the $R_{1}<R_{2}$ case, $R_{1}=20$, $R_{2}=50$, and *N* is fixed as 10,000 in both of the two cases. These values are set by referring to [8]. We use MATLAB to get the simulation results.

### 4.2. Impact of Quality of Data Rate

**Figure 4:**
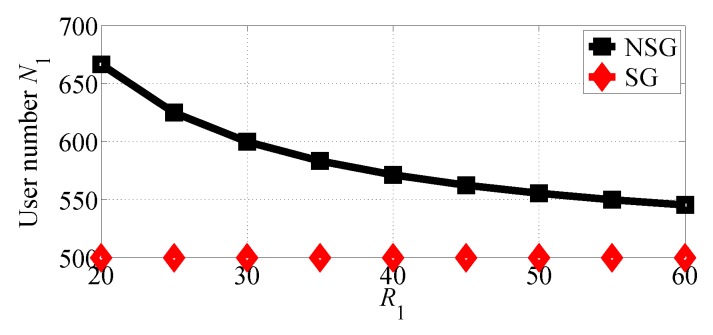
The number of users that choose SP1 with varying $R_{1}$ in the two competition scenarios for $R_{1}>R_{2}$.

**Figure 5:**
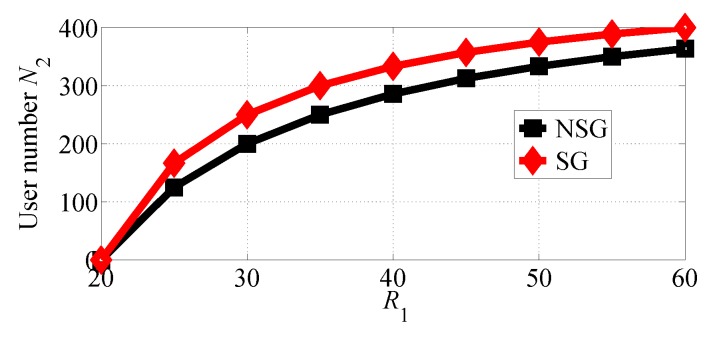
The number of users that choose SP2 with varying $R_{1}$ in the two competition scenarios for $R_{1}>R_{2}$.

**Figure 6:**
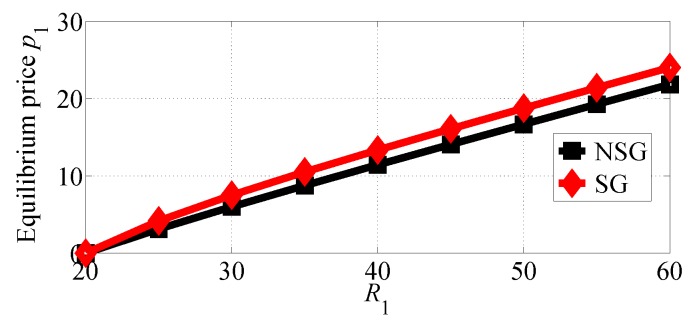
The equilibrium price of SP1 with varying $R_{1}$ in the two competition scenarios for $R_{1}>R_{2}$.

**Figure 7:**
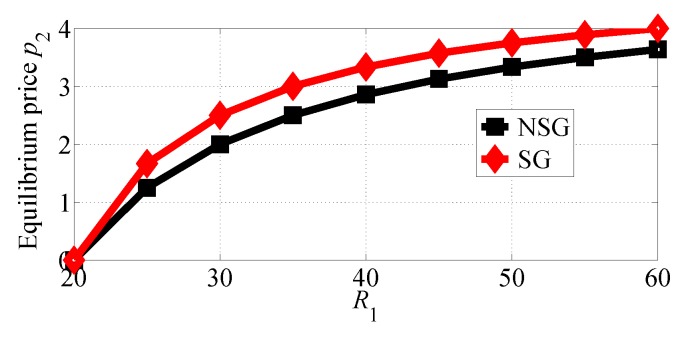
The equilibrium price of SP2 with varying $R_{1}$ in the two competition scenarios for $R_{1}>R_{2}$.

We first analyze the impact of $R_{1}$ varying in the range [20, 50] with $R_{2}=20$. Figure 4 and Figure 5 show, respectively, the number of users choosing SP1 and SP2 in the two competition scenarios vary with $R_{1}$ increasing. From the two figures we observe that SP1 can attract more users in NSG scenario than in SG scenario, while SP2 can attract more users in SG scenario than in NSG scenario. The two figures indicate that the number of users that chooses SP1 decreases in NSG scenario while the number of users that chooses SP2 increases with $R_{1}$ increasing. This is because SP1 achieves much higher equilibrium price for its services with $R_{1}$ increasing, as can be observed from Figure 6 and Figure 7. From Figure 4 and Figure 5, it is clearly observed that SP1 attracts more users than SP2 as it can provide higher quality of service.

Figure 6 and Figure 7 depict how the equilibrium prices set by SP1 and SP2, respectively, vary in the considered two competition scenarios. The results of the two figures show that both SPs can achieve higher equilibrium prices in the SG scenario than in the NSG scenario. The two figures also suggest that both SPs can set the higher equilibrium prices if $R_{1}$ increases. With comparing these two figures, we can observe that SP1 sets much higher equilibrium prices than SP2 in the two competition scenarios.

**Figure 8:**
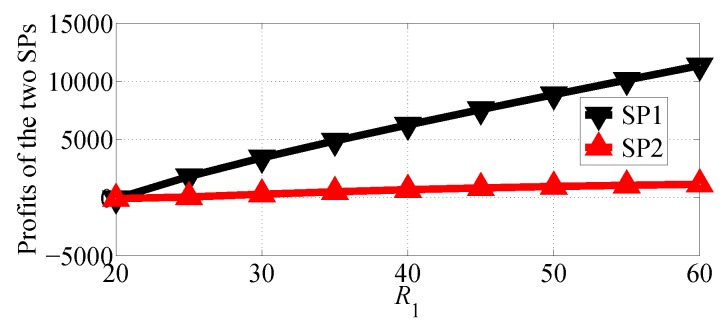
Comparing the profits of the two SPs with varying $R_{1}$ in the NSG scenario for $R_{1}>R_{2}$.

**Figure 9:**
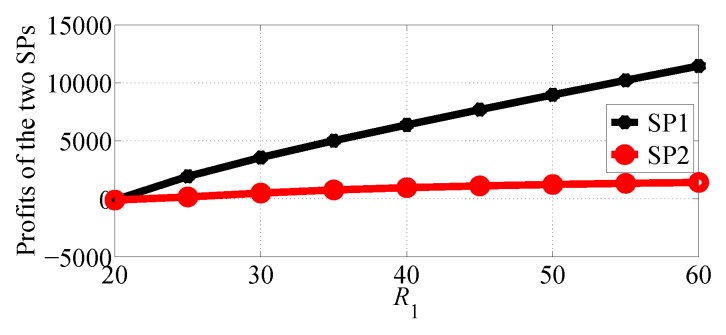
Comparing the profits of the two SPs with varying $R_{1}$ in the SG scenario for $R_{1}>R_{2}$.

In Figure 8 and Figure 9, we compare the profits of the two SPs with varying $R_{1}$ in the range [20, 60] and $R_{2}=20$ in NSG and SG scenarios, respectively. From the two figures we can observe that SP1 gets more profits than SP2 in the two competition scenarios. The two figures also show that both of SP1 and SP2 can get more profits if $R_{1}$ increases.

**Figure 10:**
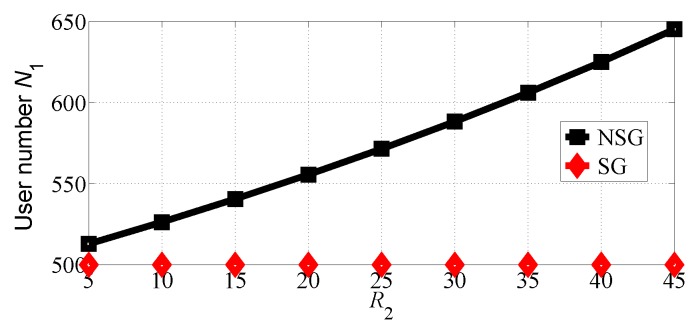
The number of users that choose SP1 with varying $R_{2}$ in the two competition scenarios for $R_{1}>R_{2}$.

**Figure 11:**
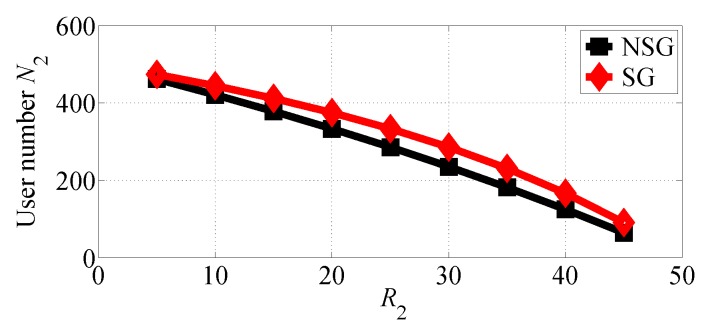
The number of users that choose SP2 with varying $R_{2}$ in the two competition scenarios for $R_{1}>R_{2}$.

**Figure 12:**
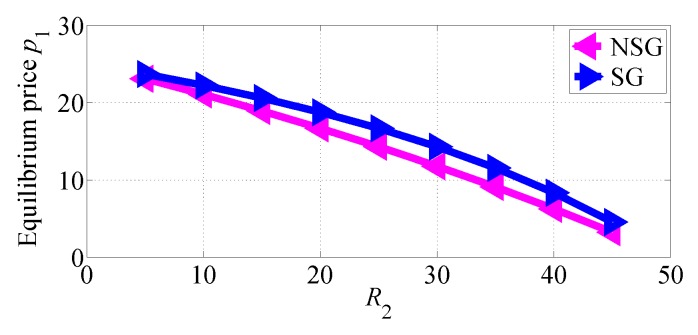
The equilibrium price of SP1 varies with $R_{2}$ increasing in the two competition scenarios for $R_{1}>R_{2}$.

We next analyze the impact of $R_{2}$ varying in the range [10, 45] with $R_{1}=50$. The number of users choosing SP1 and SP2 in the two competition scenarios is shown in Figure 10 and Figure 11, respectively. From Figure 10, we can find that SP1 attracts more users in the NSG scenario than in the SG scenario while SP2 attracts more user in the SG scenario than in the NSG scenario. We can also observe that the number of users choosing SP2 decreases even if its data rate increases. This is because more users tend to choose SP1 whose equilibrium price decreases with $R_{2}$ increasing, as illustrated in Figure 12.

**Figure 13:**
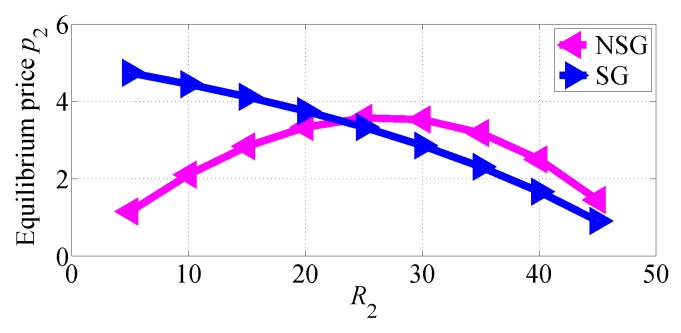
The equilibrium price of SP2 varies with $R_{2}$ increasing in the two competition scenarios for $R_{1}>R_{2}$.

In Figure 12 and Figure 13, we respectively show how the equilibrium prices set by SP1 and SP2 vary with $R_{2}$ increasing in the two competition scenarios. From Figure 12 we can observe that SP1 sets higher equilibrium price in SG scenario than in NSG scenario, and its equilibrium price decreases with $R_{2}$ increasing. From Figure 13 we can also observe that SP2 achieves higher equilibrium price in SG scenario, until reached a threshold; above this threshold, it sets higher equilibrium prices in NSG scenario. Figure 13 suggests that the equilibrium price of SP2 in NSG scenario first increases then decreases while its data rate increases.

**Figure 14:**
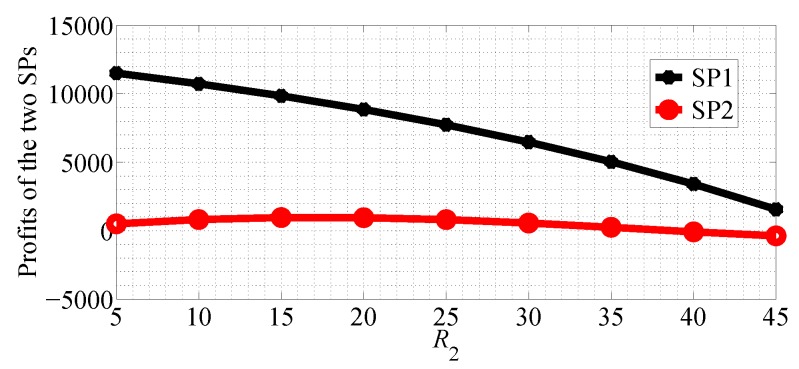
Comparing the profits of the two SPs with $R_{2}$ increasing in the NSG scenario for $R_{1}>R_{2}$.

**Figure 15:**
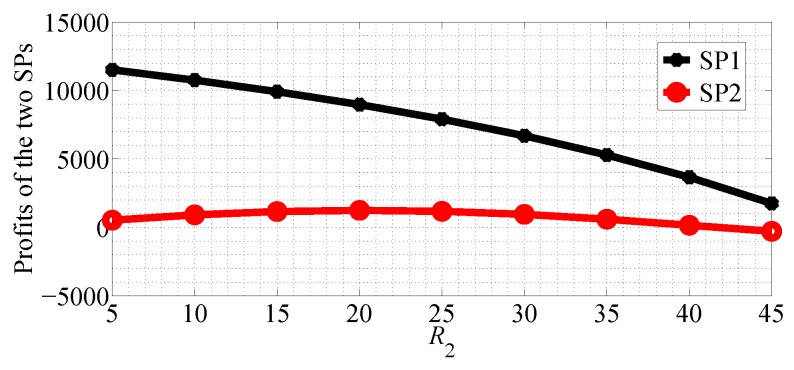
Comparing the profits of the two SPs with $R_{2}$ increasing in the SG scenario for $R_{1}>R_{2}$.

In Figure 14 and Figure 15, we compare the profits of the two SPs with $R_{2}$ varying range in [5, 45] and $R_{1}=50$ in NSG and SG scenarios, respectively. From the two figures we can observe that the profit of SP1 decreases with $R_{2}$ increasing and the profit of SP2 first increases then it decreases in the two competition scenarios.

**Figure 16:**
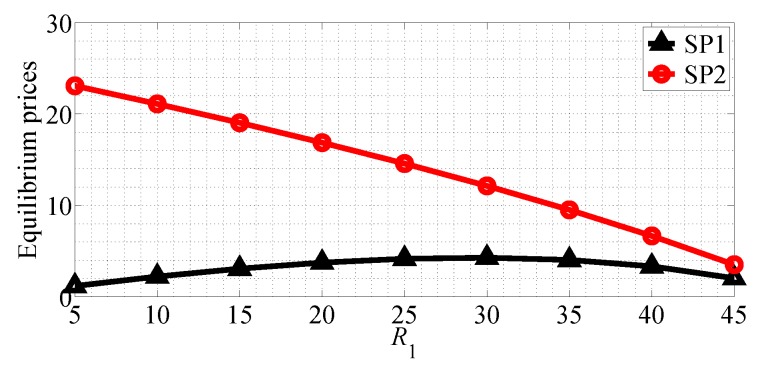
The equilibrium prices of the two SPs with varying $R_{1}$ in the SG scenario for $R_{1}<R_{2}$.

**Figure 17:**
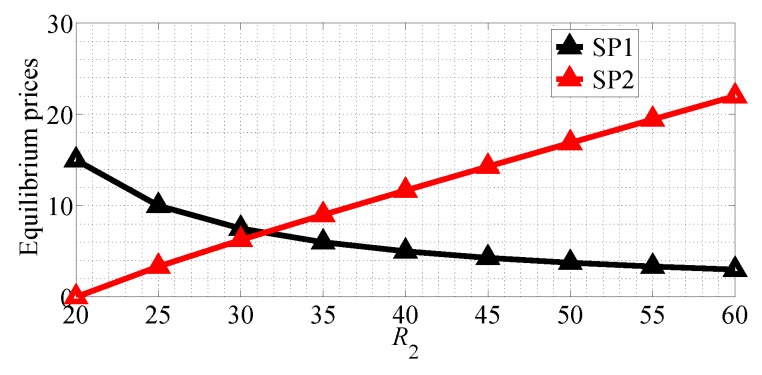
The equilibrium prices of the two SPs with varying $R_{2}$ in the SG scenario for $R_{1}<R_{2}$.

We next analyze the impact of the quality of data rate on the equilibrium prices and profits of the two SPs for $R_{1}<R_{2}$ case in SG scenario. Figure 16 shows the equilibrium price of the two SPs with $R_{1}$ varying range in [5, 45] and $R_{2}=50$ in SG scenario. From this figure we can observe that the equilibrium price of SP1 first increases then decreases with $R_{1}$ increasing, and the equilibrium price of SP2 decreases with $R_{1}$ increasing in SG scenario. Figure 17 shows the equilibrium price of the two SPs with $R_{2}$ varying range in [20, 60] and $R_{1}=20$ in SG scenario. From this figure we can observe that the equilibrium price of SP1 decreases with $R_{2}$ increasing and the equilibrium price of SP2 increases in the SG scenario. From this figure we can also observe that SP2 should provide better quality of data rate to achieve higher equilibrium price.

**Figure 18:**
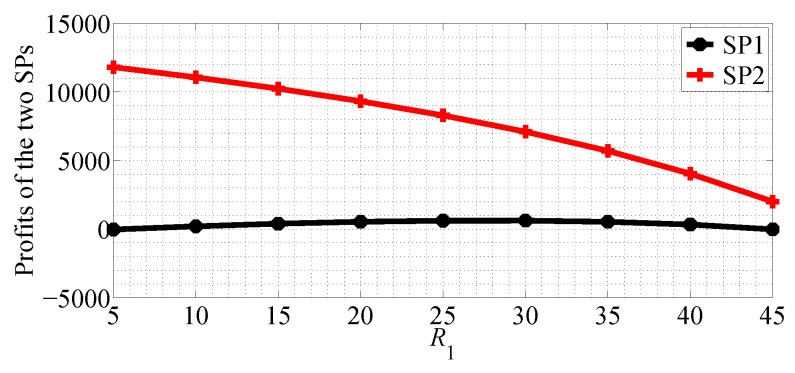
The profits of the two SPs with varying $R_{1}$ in the SG scenario for $R_{1}<R_{2}$.

**Figure 19:**
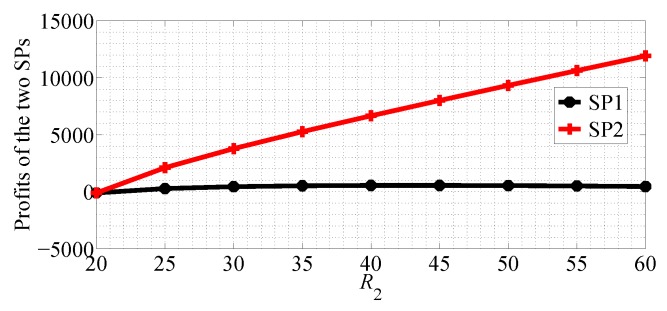
The profits of the two SPs with varying $R_{2}$ in the SG scenario for $R_{1}<R_{2}$.

In Figure 18 shows profits of the two SPs with $R_{1}$ varying range in [5, 45] and $R_{2}=50$ in SG scenario. From this figure we can observe that the profit of SP1 first increases then decreases with $R_{1}$ increasing, and the profit of SP2 decreases in SG scenario. Figure 19 shows the equilibrium prices of the two SPs with $R_{2}$ varying range in [20, 60] and $R_{1}=20$ in SG scenario. From this figure we can observe that the profit of SP1 first increases then decreases with $R_{2}$ increasing, but the change is not obvious. However, the profit of SP2 increases rapidly with $R_{2}$ increasing. From Figure 18 and Figure 19 we get the observation that the quality of data rate has more impact on the profit of SP2.

### 4.3. Impact of Cost Factor

**Figure 20:**
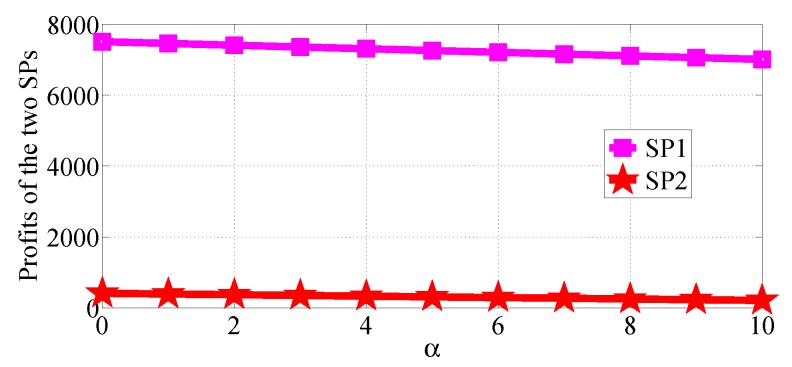
The impact of α on the profits of the two SPs in the NSG scenario for $R_{1}>R_{2}$.

**Figure 21:**
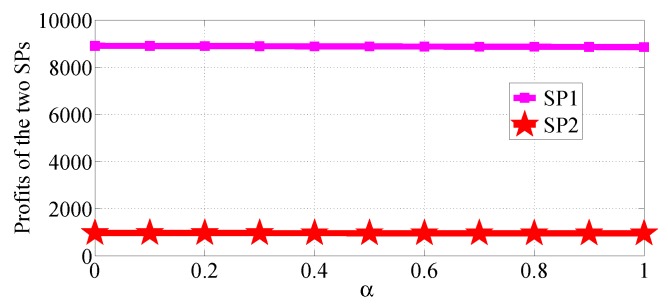
The impact of α on the profits of the two SPs in the SG scenario for $R_{1}>R_{2}$.

**Figure 22:**
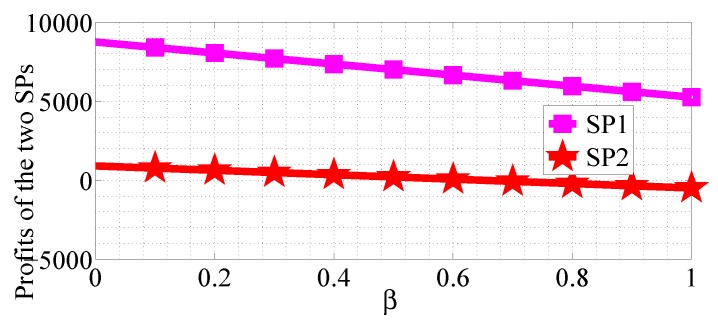
The impact of β on the profits of the two SPs in the NSG scenario for $R_{1}>R_{2}$.

**Figure 23:**
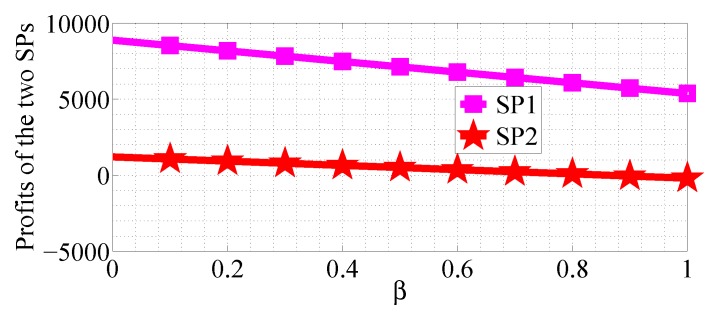
The impact of β on the profits of the two SPs in the SG scenario for $R_{1}>R_{2}$.

We analyze how the cost factors affect the profits of the two SPs in the two competition scenarios. We first consider the $R_{1}>R_{2}$ case and analyze the impact of α varying in the range [0, 10] with β=0.5, $R_{1}=50$ and $R_{2}=20$, then analyze the impact of β varying in the range [0, 1] with α=10, $R_{1}=50$ and $R_{2}=20$. Figure 20 and Figure 21 show how α affects the profits of the two SPs in NSG scenario and SG scenario, respectively. Figure 22 and Figure 23 show how β affects the profits of the two SPs with α=1, $R_{1}=50$ and $R_{2}=20$ in NSG scenario and SG scenario, respectively. By comparing the four figures we observe that the cost factor β has a higher impact on the profits of the two SPs than the cost factor of α.

**Figure 24:**
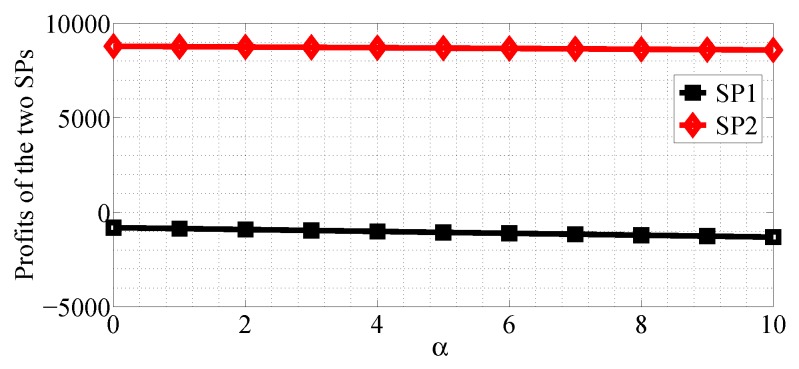
The impact of α on the profits of the two SPs in the SG scenario for $R_{1}<R_{2}$.

**Figure 25:**
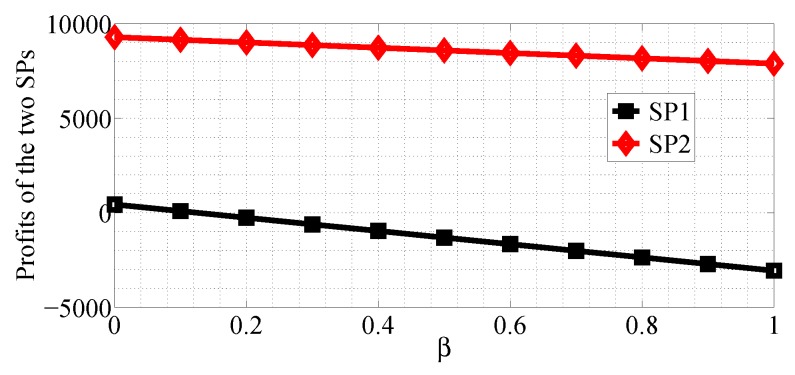
The impact of β on the profits of the two SPs in the SG scenario for $R_{1}<R_{2}$.

We next analyze how the cost factors affect the profits of the two SPs in the SP scenario for the $R_{1}<R_{2}$ case. We first analyze the impact of α varying in the range [0, 10] with β=0.5, $R_{1}=10$ and $R_{2}=50$, then analyze the impact of β varying in the range [0, 1] with α=10, $R_{1}=20$ and $R_{2}=50$. From Figure 22, Figure 23, Figure 24 and Figure 25, we can observe that the profits of the two SPs decrease with the two cost factors α and β increasing. We can also observe that β has more impact on the profits of the two SPs.

## 5. Conclusions

We studied price competition for the provision of WSN-based services in an IoT market, where two SPs compete for a common pool of end users. We modelled the interaction between the two SPs and end users as a Stackelberg game, where the two SPs set the prices for their services in the first stage, and in the second stage the end users make their decisions to buy services from one of the two SPs or choose neither of them. In particular, we studied two competition scenarios between the two SPs, i.e., the NSG scenario and SG scenario.

For the $R_{1}>R_{2}$ case, our numerical results show that as the data rate of SP1 increases, SP1 can attract more users and set higher prices in equilibrium than SP2 in the two competition scenarios, and SP1 sets higher equilibrium prices in NSG scenario than that in SG scenario while SP2 sets higher equilibrium prices in SG scenario than in NSG scenario. With the data rate of SP2 increasing, SP1 can attract more users in NSG scenario than in SG scenario while SP2 can attract more users in SG scenario than in NSG scenario. Furthermore, the equilibrium prices of SP1 decreases in the two competition scenarios and the equilibrium price of SP2 first increases and then decreases in the NSG scenario. For the $R_{1}<R_{2}$ case, our numerical results show that the increasing of $R_{1}$ does not necessary mean that SP1 can achieve higher equilibrium price. Although SP1 has first-move advantage in the SG scenario for the $R_{1}<R_{2}$ case, it does not obtain more revenue than SP2. Our numerical results on the cost factors suggest that β has more impact on the profits of the two SPs than α for both $R_{1}>R_{2}$ and $R_{1}<R_{2}$ cases.

In this paper, we focus on competition scenarios between the two SPs. In the practical case, the two SPs may cooperate with each other to improve their profits. For example, they may form a coalition to improve the profits of them. Such case is widely studied in the literature, such as [32,33]. There are several interesting research directions which can be left as future works. First, a comparison of the profits in the two competition scenarios and the cooperation case is very interesting. Second, we can extend this work to the oligopoly case where there are more than two SPs. In this case, we can apply the model in [34], where the authors studied price competition in an oligopoly network market. Third, we can extend the static scenario to the dynamic scenario where the data rate of the two SPs may change in different time slots and users may have different preferences for services in different time slots. The evolutionary game can be applied in the dynamic scenario [19].

## Abbreviations

The following abbreviations are used in this manuscript:

IoT	Internet of Things
WSNs	Wireless Sensor Networks
SPs	service providers
NSG	noncooperative strategic game
SG	Stackelberg game
WTP	willingness-to-pay

## Appendix A. Users’ Joining Decision Policies

In Proposition 1, for case (1), if $\theta_{1}>\theta^{*}$, according to Equations (7) and (8) we can get $F_{1}\;=\;1-F_{1}\left(\theta_{1}\right)=0$, $F_{2}=0$. For case (2), if $\theta_{1}<\theta^{*}$, which means that

(A1) $$\theta_{1}-\theta^{*}=\frac{R_{1}p_{2}-R_{2}p_{1}}{R_{1}\left(R_{1}-R_{2}\right)}<0,$$

As $R_{1}-R_{2}>0$, from Equation (A1) we get $R_{1}p_{2}-R_{2}p_{1}<0$.

From

(A2) $$\theta_{2}-\theta^{*}=\frac{R_{1}p_{2}-R_{2}p_{1}}{R_{2}\left(R_{1}-R_{2}\right)},$$

(A3) $$\theta_{1}-\theta_{2}=\frac{R_{2}p_{1}-R_{1}p_{2}}{R_{1}R_{2}}.$$

As $R_{1}p_{2}-R_{2}p_{1}<0$, we get $\theta_{2}-\theta^{*}<0$ and $\theta_{1}-\theta_{2}>0$, that is, $\theta_{2}<\theta_{1}<\theta^{*}$. According to Equations (7) and (8) we have

(A4) $$F_{1}=1-F\left(\theta_{1}\right),$$

(A5) $$F_{2}=F\left(\theta^{*}\right)-F\left(\theta_{2}\right).$$

## Appendix B. Proof of Proposition 2

The objective function of Problem1 in Equation (15) is a convex function, therefore, by taking the derivative of $\pi_{1}$ with respective to $p_{1}$, and setting the it to zero,

(A6) $$\frac{\partial \pi_{1}}{\partial p_{1}}=1-\frac{2p_{1}-p_{2}}{R_{1}-R_{2}}=0,$$

From which, we get

(A7) $$p_{1}=\frac{R_{1}-R_{2}+p_{2}}{2}.$$

Similarly, by taking the derivative of $\pi_{2}$ with respective to $p_{2}$, and setting the equality to zero, we have

(A8) $$\frac{\partial \pi_{2}}{\partial p_{2}}=\frac{p_{1}-2p_{2}}{R_{1}-R_{2}}-\frac{2p_{2}}{R_{2}}=0,$$

From which, we have

(A9) $$p_{2}=\frac{p_{1}R_{2}}{2R_{1}}.$$

By solving Equations (A7) and (A9), we get the optimal subscription prices of the two SPs in the NSG scenario, which are denoted respectively as

(A10) $$p_{1}^{n}=\frac{2R_{1}\left(R_{1}-R_{2}\right)}{4R_{1}-R_{2}},$$

(A11) $$p_{2}^{n}=\frac{R_{2}\left(R_{1}-R_{2}\right)}{4R_{1}-R_{2}}.$$

Accordingly, by substituting Equations (A10) and (A11) into Equations (9) and (10) respectively, the number of users that choose of SP1 and SP2 in NSG scenario are denoted respectively as

(A12) $$N_{1}^{n}=NF_{1}=N\frac{2R_{1}}{4R_{1}-R_{2}},$$

(A13) $$N_{2}^{n}=NF_{2}=N\frac{2(R_{1}-R_{2})}{4R_{1}-R_{2}}.$$

## Appendix C. Proof of Proposition 3

The objective function of Equation (21) is convex, from which the optimal subscription price $p_{2}$ can be expressed as function of $p_{1}$ as follows

(A14) $$p_{2}=\frac{p_{1}R_{2}}{2R_{1}},$$

By inserting the above equation into the objective function of and Equation (22), we get an equivalent problem **Problem5:**

(A15) $$\begin{array}{c} \max_{p_{1}}N\left[1-\frac{\left(2R_{1}-R_{2}\right)p_{1}}{2R_{1}\left(R_{1}-R_{2}\right)}\right]p_{1}-R_{1}\left[\alpha +\beta \left(R_{1}+R_{2}\right)\right]\\ s.t.\;p_{1}\geq 0 \end{array}$$

It is obvious that the objective function of Equation (A15) is convex, therefore, from the first-order condition, the optimal subscription price of SP1 in SG scenario denoted by $p_{1}^{s}$ is expressed as

(A16) $$\begin{array}{c} p_{1}^{s}=\frac{R_{1}\left(R_{1}-R_{2}\right)}{2R_{1}-R_{2}}, \end{array}$$

By substituting Equation (A16) into Equation (A14) the optimal subscription price of SP2 in SG scenario (denoted by $p_{2}^{s}$) is

(A17) $$p_{2}^{s}=\frac{R_{2}\left(R_{1}-R_{2}\right)}{2(2R_{1}-R_{2})}.$$

Substituting Equations (A16) and (A17) into Equations (9) and (10), we obtain

(A18) $$N_{1}^{s}=NF_{1}=\frac{N}{2},$$

(A19) $$N_{2}^{s}=NF_{2}=N\frac{R_{1}-R_{2}}{2R_{1}-R_{2}}.$$

## Appendix D. Proof of Proposition 4

For the $R_{1}<R_{2}$ case, the objective functions are the same as the $R_{1}<R_{2}$ case, but $N_{1}^{s}$ and $N_{2}^{s}$ are respectively denoted in Equations (11) and (12).

The objective function of Equation (21) is convex, from which the optimal subscription price $p_{2}$ can be expressed as function of $p_{1}$ as follows

(A20) $$p_{2}=\frac{p_{1}-R_{1}+R_{2}}{2},$$

By inserting the above equation into the objective function of and Equation (22), we get an equivalent problem **Problem6:**

(A21) $$\begin{array}{c} \max_{p_{1}}N\left[\frac{p_{1}+R_{1}-R_{2}}{2(R_{1}-R_{2})}-\frac{p_{1}}{R_{1}}\right]p_{1}-R_{1}\left[\alpha +\beta \left(R_{1}+R_{2}\right)\right]\\ s.t.\;p_{1}\geq 0 \end{array}$$

It is obvious that the objective function of Equation (A21) is convex, therefore, from the first-order condition, the optimal subscription price of SP1 in SG scenario denoted by $p_{1}^{s2}$ is expressed as

(A22) $$\begin{array}{c} p_{1}^{s2}=\frac{R_{1}\left(R_{2}-R_{1}\right)}{2(2R_{2}-R_{1})}, \end{array}$$

By substituting Equation (A22) into Equation (A20) the optimal subscription price of SP2 in SG scenario (denoted by $p_{2}^{s2}$) is

(A23) $$p_{2}^{s2}=\frac{\left(4R_{2}-R_{1}\right)\left(R_{2}-R_{1}\right)}{4(2R_{2}-R_{1})}.$$

Substituting Equations (A22) and (A23) into Equations (11) and (12), we obtain

(A24) $$N_{1}^{s2}=NF_{1}=\frac{N}{4},$$

(A25) $$N_{2}^{s2}=NF_{2}=N\frac{4R_{2}-R_{1}}{4(2R_{2}-R_{1})}.$$
